# Effects of organic acid-preserved cereal grains in sow diets during late gestation and lactation on the performance and faecal microbiota of sows and their offspring

**DOI:** 10.1186/s40104-025-01171-3

**Published:** 2025-03-12

**Authors:** Shane Maher, Torres Sweeney, Stafford Vigors, Michael McDonald, John V. O’Doherty

**Affiliations:** 1 School of Agriculture and Food Science, University College Dublin, Dublin 4, Ireland; 2 School of Veterinary Medicine, University College Dublin, Dublin 4, Ireland

**Keywords:** Cereal preservation, Lactation feeding, Microbiota, Nutrient digestibility, Offspring, Organic acids, Sows

## Abstract

**Background:**

Organic acids (OA) and maternal nutritional strategies have been demonstrated to promote piglet health and development. The objective of this study was to investigate the effects of incorporating OA-preserved cereal grains into sow diets during late gestation and lactation, aiming to reduce the metabolic demands of lactation while optimising offspring development and growth until slaughter. The experiment compared OA-preserved wheat and barley to conventionally dried grains, focusing on sow and offspring performance, as well as their faecal microbiota during lactation. Forty sows were blocked based on parity, body weight and back fat thickness on d 100 of gestation and assigned to one of two diets: a dried grain lactation diet and a preserved grain lactation diet. Sow faecal samples were collected at farrowing for the coefficient of apparent total tract digestibility (CATTD) of nutrients and microbial analysis. Offspring faecal samples were collected on d 10 postpartum and at weaning (d 26 postpartum) for microbial analysis.

**Results:**

Sow body weight, back fat changes, gestation and lactation length, total piglets born, wean-to-oestrus interval, and lactation efficiency were unaffected by sow diet (*P* > 0.05). However, sows offered the preserved grain diet exhibited improved CATTD of dry matter, nitrogen, gross energy, and neutral detergent fibre (*P* < 0.05). While no maternal effect was observed on offspring growth during lactation (*P* > 0.05), pigs from sows offered the preserved grain diet showed improved growth and feed efficiency from weaning until slaughter (d 168) compared to those from sows offered the dried grain diet (*P* < 0.05). The preserved grain diet also reduced the abundance of Proteobacteria in sow faeces at farrowing and in their offspring on d 10 postpartum, and improved piglet faecal scores throughout lactation (*P* = 0.05). At weaning, piglets from sows offered the preserved grain diet exhibited an increased abundance of *Lactobacillus* and reduced abundance of *Alistipes* in their faeces (*P* < 0.05).

**Conclusion:**

OA-preserved grains enhanced the CATTD of nutrients in sows, promoted healthier piglet faecal scores during lactation, and improved offspring growth performance post-weaning, potentially linked to beneficial changes observed in the faecal microbiota of sows and their offspring during lactation.

**Supplementary Information:**

The online version contains supplementary material available at 10.1186/s40104-025-01171-3.

## Background

Continuous genetic selection for enhanced pig performance traits has resulted in larger litters, with fast-growing, high-lean progeny [1]. However, these improvements have also been associated with increased metabolic and nutritional demands of sows [2, 3], along with a higher incidence of low birth weight piglets, greater within-litter variation and increased piglet mortality [4–6]. At weaning, the dietary transition from a liquid milk-based diet to a solid cereal-based diet challenges the immature digestive capability of piglets. Concurrently, the abrupt separation from the sow and the restructuring of group pen dynamics amplify pathogen exposure and social stress [7]. This sequence of events results in a transient reduction in energy intake and growth, as well as gastrointestinal disturbances post-weaning (PW) [8–10]. These challenges are now further exacerbated by new restrictions on in-feed antimicrobials and zinc oxide in pig diets [11].

Cereal grains are the primary energy source in swine feed formulations, constituting a large proportion of the diet. In the absence of in-feed antimicrobials, maintaining grain quality, consistency, and nutrient composition is crucial for optimising feed efficiency and mitigating health risks associated with nutrient deficiencies and contaminants such as mycotoxins [12, 13]. The post-harvest storage period poses a significant threat to grain quality; thus, effective preservation methods must be implemented [14]. Mechanically drying cereal grains to a moisture content below 140 g/kg is a conventional preservation technique in temperate climates to avoid deterioration during storage [15]. However, the increasing financial and environmental implications associated with drying have resulted in alternative preservation technologies being explored [16–18].

Organic acids (OA) and their salts are commonly used food preservatives due to their antifungal and antibacterial properties [19, 20]. Increasing evidence suggests that dietary OA supplementation could support the transition to antimicrobial-free feeding, as recently reviewed by several authors [21–25]. Supplementing feed or drinking water with OA can improve nutrient digestibility and promote eubiosis, enhancing pig immunity and growth performance [21, 23, 26]. Recent studies have demonstrated that incorporating cereal grains preserved with OA blends can enhance nutrient digestibility, gut health, and growth performance during the post-weaner stage compared to conventionally dried grain [27, 28], offering a practical and potentially more sustainable approach to OA supplementation.

Implementing nutritional strategies during late gestation and lactation can enhance sow metabolic and health status, leading to healthier piglets and reduced reliance on antimicrobials [29, 30]. Furthermore, the sow is the primary contributor to the establishment of her offspring’s gut microbiota [31, 32]. Maternal strategies that can influence the acquisition of beneficial bacteria could further promote piglet development and growth [33]. Previous research has shown that supplementating sows with OA during late gestation and lactation can modulate microbial populations and improve offspring growth performance [34–36]. However, a contextual gap exists in the current literature on the potential benefits of incorporating OA-preserved grain into sow diets. Thus, the objectives of this study were to compare the nutritional quality of OA-preserved wheat and barley with conventionally dried grain and to investigate the effects of incorporating these grains into sow diets during late gestation and lactation. It was hypothesised that offering sows OA-preserved grain diets would improve nutrient digestibility, minimise body condition changes, and modulate their faecal microbiota, thereby enhancing the development and growth of their offspring from birth to slaughter.

## Materials and methods

### Grain management and quality analysis

The cereal grains used in this study were established in Ireland during the 2022 growing season and included a winter wheat variety (*JB Diego*) and a spring barley variety (*SY Errigal*) sourced from Platin Grain (Drogheda, Louth, Ireland). The wheat received a 3-spray fungicide programme and a 3-split nitrogen (N) application totalling 180 kg N/ha. The barley crop received a 2-spray fungicide programme and a 2-split N application totalling 140 kg/ha. The wheat and barley were harvested in August 2022 at a moisture content of 179.7 g/kg and 182.1 g/kg, respectively. Before storage, both crops were split into two batches and were either dried or preserved with an organic acid mould inhibitor, as previously described by Maher et al. [27]. Briefly, one batch was dried for 3 h using a continuous flow-type grain dryer (Cimbria, Thisted, Denmark) until a moisture content of 140 g/kg was achieved. The other batch received a topical administration of an OA liquid surfactant mould inhibitor (MycoCURB ES Liquid) sourced from Adesco Nutricines (Dungarvan, Waterford, Ireland). This mould inhibitor contained 650 g/kg propionic acid, 70 g/kg ammonium propionate, 17.5 g/kg glycerol polyethylene glycol ricinoleate, and a carrier, and was applied at a rate of 4 g/kg. Grains were ventilated and stored for 100 d before feed manufacture. At harvest, the moisture content of the wheat and barley was analysed using a DICKEY-john GAC 2500-UGMA electronic moisture metre (Auburn, IL, USA). A Pfeuffer Chondrometer and bulk density calibration chart were used to measure grain density and thousand-grain weight was assessed by recording the weight of 1,000 grains using a Pfeuffer Contador seed counter (Kitzingen, Germany). Representative grain samples were collected after storage and were analysed for dry matter (DM), ash, gross energy (GE), crude protein (CP), crude fibre, starch, ether extract, pH, total mould count (TMC), and mycotoxins. The crude ash content was determined after ignition of a weighted sample in a muffle furnace (Nabertherm) at 550 °C for 6 h. The GE content was determined using an adiabatic bomb calorimeter (Parr Instruments, St, Moline, IL, USA). The CP (N × 6.25) content was determined using the LECO FP 528 instrument (Leco Instruments, Stockport, UK). The crude fibre content was determined using the Ankom 220 Fibre Analyser (Ankom Technology, NY, USA) according to the AOAC.962.09 [37]. Starch concentration was determined using a Megazyme assay kit (Megazyme Int., Wicklow, Ireland). Ether extract concentration was determined using light petroleum ether and Soxtec instrumentation (Tecator, Sweden) according to the AOAC.920.39 [37]. Grain pH was measured using a pH probe (Mettler-Toledo FiveEasy Plus; Greifensee, Switzerland), calibrated using certified pH 4 and pH 7 buffer solutions. The TMC of the grain was determined by the colony count technique (ISO21527-2:2008) as described by Laca et al. [38]. The mycotoxin presence of aflatoxin B_1_, B_2_, G_1_ and G_2_, fumonisin B_1_ and B_2_, deoxynivalenol (DON), T-2 Toxin, HT-2 Toxin, zearalenone (ZEN) and ochratoxin A (OTA) were determined by liquid chromatography-mass spectrometry [39]. The chemical and mycotoxin analyses of the grains are presented in Table 1.

**Table 1:** The chemical analysis of the wheat and barley included in the sow diets on a dry matter basis, g/kg DM unless otherwise stated

Crop type	Wheat		Barley	
**Grain preservation**	**Dried**	**Preserved**	**Dried**	**Preserved**
Dry matter, g/kg	868.4	820.3	872.1	817.9
Ash	17.5	17.3	22.7	21.6
Gross energy, MJ/kg DM	18.0	18.0	17.4	17.3
Ether extract	16.9	16.8	21.8	20.8
Crude protein	112.9	112.7	106.6	107.3
Crude fibre	27.1	26.2	57.9	55.6
Starch	670.9	666.8	612.5	610.0
pH	6.0	5.7	5.8	5.6
Total mould count, CFU/g^a^	2.2	0.9	3.3	1.2

All mycotoxins analysed were below the detectable levels: Aflatoxin B_1_, B_2_, G_1_and G_2_(< 1 μg/kg); Fumonisin B_1_(< 125 μg/kg) and Fumonisin B_2_(< 50 μg/kg) Deoxynivalenol (< 75.0 μg/kg), HT-2 Toxin (< 4.0 μg/kg), T-2 Toxin (< 4.0 μg/kg), Zearalenone (< 10.0 μg/kg) and Ochratoxin A (< 1.0 μg/kg)

^a^These values were log-transformed

### Experimental design

A total of 40 sows (Large White × Landrace) were selected on d 100 of gestation and blocked according to parity (mean ± SD; 3.2 ± 0.2), body weight (BW; 272.4 kg ± 4.5) and back fat thickness (BF; 16.0 mm ± 0.3). The sow parity distribution ranged from 20% at parity 0, 19% at parity 1, 18% at parity 2, and 15% at parity 3, with 28% in parities 4 and 5 combined. Within each block, sows were assigned to one of two dietary groups: dried grain lactation diet and preserved grain lactation diet (*n* = 20). Sows remained on their allocated diets until the subsequent service (d 30 postpartum). The ingredient composition of the diet is presented in Table 2.

**Table 2:** The ingredient composition of the experimental sow diets

Ingredients	g/kg
Wheat^a^	380
Barley^a^	250
Soyabean meal	170
Soya hulls	10
Full-fat soya	80
Soya oil	25
Pollard	40
Beet pulp	10
Salt	5
Mono calcium phosphate	8
Calcium carbonate (limestone)	12
L-Lysine HCl (78.8%)	4
DL-Methionine	1.3
L-Threonine	2.5
L-Tryptophan	0.7
Premix concentrate^b^	1.5^a^

^a^Grain was either mechanically dried to a moisture content of 140 g/kg or preserved with an organic acid mould inhibitor at an inclusion rate of 4 g/kg and remained at 180 g/kg moisture content

^b^Vitamin and mineral premix (per kg lactation diet): 70 mg of Fe as FeSO_4_; 60 mg of Mn as MnO; 80 mg of Zn as ZnO; 15 mg of Cu as CuSO_4_; 0.6 mg of I as calcium iodate on a calcium sulphate/calcium carbonate carrier; 0.2 mg Se as sodium selenite; 3.4 mg of vitamin A as retinyl acetate; 25 mg of vitamin D_3_ as cholecalciferol; 100 mg of vitamin E as DL-α-tocopheryl acetate; 2 mg of vitamin K as phytylmenaquinone,, 2 mg of vitamin B_1_ as thiamine, 5 mg of vitamin B_2_ as riboflavin, 3 mg of vitamin B_6_ as pyridoxine, 0.015 mg of vitamin B_12_ as cyanocobalamin, 12 mg of nicotinic acid; 10 mg of pantothenic acid; 500 mg of choline chloride; 0.02 mg of biotin, 5 mg of folic acid

### Sow management

From d 0 to 100 of gestation, sows were managed in dynamic groups of 20 animals per pen. The pen had fully slatted floors and insulated concrete lying bays. The temperature in the gestation house was maintained at 20 °C. Sows were allocated 2.4 kg/d of a standard gestation diet in meal form via a shared trough (20 feeding places) in two equal meals. The gestation diet was formulated to contain 150 g of CP/kg, 12.2 MJ of digestible energy (DE)/kg, 8.6 MJ of net energy (NE)/kg and 6 g of standardised ileal digestible (SID) lysine/kg. From d 100 to 110 of gestation, the dynamic groups were halved and sows were penned in groups of 10 according to their allocated dietary group, with access to individual feeding crates at scheduled feeding times. They were offered 2.8 kg/d of their respective experimental diets in meal form, formulated to contain 170 g of CP/kg, 14.2 MJ of DE/kg, 10.0 MJ of NE/kg and 10 g of SID lysine/kg. Celite (1g/kg) was added to each diet to measure the coefficient of apparent total tract digestibility (CATTD) of nutrients.

On d 110 of gestation, sows were moved to individual farrowing pens (pen dimensions: 2.2 m × 2.4 m) equipped with crates, slatted floors, and heat pads for piglets (Big Dutchman, Vechta, Germany). The dietary groups were evenly dispersed across two farrowing rooms, each accommodating 20 sows per room. From d 110 until farrowing, sows were allocated a feed allowance of 2.6 kg/d. In the farrowing house, sows were fed in four equal meals through a computerised feed delivery system (HydroAir, Big Dutchman, Vechta, Germany) which recorded daily feed intake throughout lactation. Feed supply increased by 1.0 kg/d until 3 d post-farrowing and by 0.5 kg/d from then until 6 d post-farrowing. Feed curves were individually adjusted, as required, to prevent feed wastage and ensure feed intake was as close to ad libitum feeding as possible. The farrowing house temperature was maintained at approximately 24 °C and gradually reduced to 20 °C by d 6 of lactation. During the weaning-to-oestrus interval sows were relocated to the service house in groups of 10 and received 3.5 kg/d of their respective experimental diets, followed by a standard gestation diet post-service. Water was made available to sows ad libitum via single-bite drinkers throughout the experiment.

### Sow body weight and back fat thickness

Sow BW and BF were recorded on d 100 of gestation and again at weaning. Sow BW was recorded using an electronic scale (Avery, Smethwick, UK) and BF was measured using a digital back fat indicator (Renco Lean-Meter; Renco Corporation, Golden Valley, MN, USA) by placing the indicator probe on the sow’s back at the level of the second last rib, approximately 6.5 cm from the side of the spinal column. A reading from both the left and right side of the sow’s back was taken and the average of both readings was recorded [40].

### Lactation feed and energy intake, and lactation efficiency

Individual sow feed intake during lactation was recorded daily, allowing for ADFI to be calculated. Lactation dry matter intake (DMI) was also calculated using the DM of the diets. The average DE and NE intake were determined using the analysed DE and calculated NE values, respectively. Sow lactation efficiency was individually calculated from parturition to weaning and was expressed as the ratio of litter growth (total litter weight gain during lactation, g) to the energy intake of sows (total energy intake during lactation, MJ DE)[41].

### Coefficient of apparent total tract digestibility

Faecal samples were collected from 10 sows per dietary group before the expected farrowing date (between d 112 and 115 of gestation) and frozen at −20 °C for the determination of the CATTD of nutrients. Before analysis, faecal samples were dried at 55 °C for 72 h. The CATTD was calculated using the acid-insoluble ash (AIA) technique [42] and the following equation: CATTD of nutrient = 1 – (nutrient in faeces/nutrient in diet) × (AIA-diet/AIA-faeces), where nutrient in faeces and nutrient in diet represent the nutrient concentration (g/kg) in the faeces and diet DM, respectively and AIA-diet and AIA-faeces represent the marker concentrations (g/kg) in the diet and faeces DM, respectively [43].

### Pre-weaning offspring management

All farrowings were supervised, with minimal interference to avoid disruption. The number of piglets born per litter was recorded, including total-born, live-born, stillborn, and mummified piglets. When possible, litter size was standardised by cross-fostering within maternal dietary group during the first 24 h postpartum. Piglets had their tails docked, teeth clipped and received an intramuscular injection of iron (Gleptosil, Ceva Sante Animale; Lisbourne, France) within the first 5d postpartum. On d 10 postpartum, a dry pelleted starter diet (2 mm in diameter) was introduced to all farrowing pens using circular hopper creep feeders (Mini Hopper Creep Feeders, Rotecna, Spain). To minimise wastage and encourage intake, the starter diet was provided in small quantities frequently throughout the day. All feed supplied and removed was recorded to calculate litter intake from d 10 until weaning (d 26 postpartum). The starter diet was formulated to contain 196 g of CP/kg, 17.0 MJ of DE/kg, 12.0 MJ of NE/kg and 12.5 g of SID lysine/kg. To monitor the maternal effect on growth performance throughout lactation, litter size and litter weight were recorded after cross-fostering (d 0), before creep introduction (d 10) and at weaning (d 26). This data was used to determine litter gain, mean piglet weight, piglet average daily gain (ADG) and pre-weaning piglet mortality. Faecal consistency scores were determined for piglets on d 7, 14, 21 and 26 postpartum. Scoring was carried out on each farrowing pen by the same individual using a scale from 1–5 where; 1 = hard, firm faeces; 2 = slightly soft faeces; 3 = soft, partially formed faeces; 4 = loose, semi-liquid faeces and 5 = watery, mucous-like faeces [44].

### Post-weaning offspring management

To assess the residual maternal dietary effects on offspring lifetime growth and feed efficiency, 560 pigs, representing 95% of the pre-weaning population, were selected at weaning and monitored until slaughter on d 168. The remaining 5% were excluded from the study due to factors such as low weaning weight, injuries, and severe diarrhoea. Within maternal groups, the selected pigs were housed sequentially in weaner accommodations (d 26–88) and finisher accommodations (d 88–168). They were organised into mixed-sex groups of 28 animals per pen, with each pen comprised of piglets from two different sow litters (*n* = 10). Pen weight was recorded at weaning and slaughter using an electronic scale (Avery, Smethwick, UK) to calculate pig mean BW and overall ADG. Pigs remained on their starter diets until d 40 and subsequently received the following sequence of dry pelleted diets (3 mm in diameter): a two-stage weaner diet from d 40 to 56 (192 g CP/kg, 16.0 MJ DE/kg, 11.3 MJ of NE/kg and 12.5 g SID lysine/kg) and d 56 to 88 (190 g CP/kg, 15.0 MJ DE/kg, 10.6 MJ of NE/kg and 11 g SID lysine/kg) and a two-stage finisher diet from d 88 to 128 (140 g CP/kg, 14.0 MJ DE/kg, 9.9 MJ of NE/kg and 10 g SID lysine/kg) and d 128 to 168 (135 g CP/kg, 13.8 MJ DE/kg, 9.7 MJ of NE/kg and 8.5 g SID lysine/kg). All diets were formulated to meet the NRC recommendations [45].

Feed consumed per pen was recorded weekly to calculate overall average daily feed intake (ADFI) and feed conversion ratio (FCR). The temperature in the weaner accommodation was maintained at 28 °C during the first week PW and reduced by 2 °C each week until 22 °C was reached by d 56. The temperature was maintained between 20–22 °C thereafter. Ventilation for all houses was via a punched ceiling with air exhausted through a variable speed fan linked to a computer-controlled thermostat (Big Dutchman 135, Vechta, Germany). Pigs were monitored twice daily and any pig showing signs of illness was recorded and treated as per veterinary recommendations. No mixing of pigs occurred throughout the experiment. All accommodation was illuminated by daylight and artificial light. Water was available to pigs ad libitum from drinker bowls throughout the experimental period.

### Microbiological analysis

#### Sample collection

On the expected date of farrowing (d 115 of gestation), fresh sow faecal samples were collected from 10 sows per dietary group. Three piglets from the selected sows were identified based on average birth weight and a pooled faecal sample was collected on d 10 postpartum and again at weaning. Only faecal samples that had not come into contact with the pen floor were collected in sterile containers (Sarstedt, Wexford, Ireland) before being snap-frozen on dry ice and stored at −80 °C until DNA extraction for microbial analysis.

#### Microbial DNA extraction

A QIAamp PowerFecal Pro DNA Kit (Qiagen, West Sussex, UK) was used to extract the faecal microbial DNA following the manufacturer’s instructions. The quality and quantity of DNA was assessed using a Nanodrop ND-1000 Spectrophotometer (Thermo Scientific).

#### Illumina sequencing

High throughput sequencing of the V3–V5 hypervariable region of the bacterial 16S rRNA gene was performed on an Illumina MiSeq platform according to standard protocols (Eurofins Genomics, Ebersberg, Germany). The V3–V5 region was PCR-amplified using universal primers containing adapter overhang nucleotide sequences for forward and reverse index primers. Amplicons were purified using AMPure XP beads (Beckman Coulter, Indianapolis, Indiana, USA) and set up for the index PCR with Nextera XT index primers (Illumina, San Diego, California, USA). The indexed samples were purified using AMPure XP beads, quantified using a fragment analyser (Agilent, Santa Clara, California, USA) and equal quantities from each sample were pooled. The resulting pooled library was quantified using the Bioanalyser 7500 DNA kit (Agilent) and sequenced using the v3 chemistry (2 × 300 bp paired-end reads).

#### Bioinformatics

Eurofins Genomics (Germany) conducted the bioinformatic analysis of the sequences using the Quantitative Insights into Microbial Ecology package (Version 1.9.1) [46]. All raw reads passing the standard Illumina chastity filter were demultiplexed in accordance with their index sequences (read quality score > 30). The primer sequences were clipped from the beginning of the raw forward and reverse reads. If primer sequences were not perfectly matched, read pairs were eliminated to retain only high-quality reads. Paired-end reads were then merged to obtain a single, longer read that covered the entire target region using the software FLASH 2.200 [47]. The pairs were merged with a minimum overlap size of 10 bp to reduce false-positive merges. The forward read was only retained for the subsequent assessment steps when merging was not possible. Quality filtration of merged reads was then carried out according to the expected and known length variations in the V3–V5 region (ca. 535 bp). The ends of retained forward reads were clipped to a total read length of 285 bp to eliminate low-quality bases. Merged and retained reads comprising ambiguous bases were removed. The filtered reads were then used for profiling the microbiome. Chimeric reads were detected and removed based on the de-novo algorithm of UCHIME [48] as implemented in the VSEARCH package [49]. The remaining set of high-quality reads was processed using minimum entropy decomposition to partition reads into operational taxonomic units (OTU) [50, 51]. The DC-MEGABLAST alignments of cluster representative sequences to the NCBI nucleotide sequence database were conducted for the taxonomic assignment of every OTU. A sequence identity of 70% across at least 80% of the representative sequence was the minimum requirement for evaluating reference sequences. Abundances of bacterial taxonomic units were normalised using linear-specific copy numbers of the appropriate marker genes to enhance estimates [52]. The normalised OTU table combined with the phenotype metadata and phylogenetic tree comprised the data matrix. The data matrix was loaded into the phyloseq package in R (Version 3.5.0, accessed on 14/03/2024). The dynamics of richness and diversity were computed with the Observed species, Shannon, Simpson, and Fisher indices. The Shannon and Simpson indices accounted for richness and evenness parameters. Additionally, beta diversity was estimated by normalising the data to produce taxonomic feature counts that were comparable across all samples. Several distance metrics were considered to calculate the distance matrix of the various multidimensional reduction processes including weighted and unweighted UniFrac distance and non-phylogenetic distance metrics (Bray–Curtis, Jensen-Shannon divergence and Euclidian) using phyloseq in R as previously described by Dowley et al. [53]. Differential abundance testing was carried out on tables extracted from the phyloseq object at the phylum, family and genus level.

### Feed analysis

Representative feed samples were collected at regular intervals and retained for chemical and mycotoxin analyses. The feed was milled through a 1-mm screen (Christy and Norris Hammer Mill, Chelmsford, UK) and analysed for DM, ash, GE, ether extract, CP, crude fibre, neutral detergent fibre (aNDF), acid detergent fibre (ADF), starch, total mould count (TMC), and mycotoxins, as previously described. The aNDF and ADF were determined using the Ankom 220 Fibre Analyser according to the method of Mertens et al. (method 2002.04) [54]. The chemical and mycotoxin analyses of the sow diets are presented in Table 3.

**Table 3:** The chemical analysis of the experimental sow diets on an as-fed basis, g/kg unless otherwise stated

Grain preservation method	Dried	Preserved
Chemical composition, g/kg		
Dry matter	885.3	867.2
Ash	52.8	50.8
Gross energy, MJ/kg	16.1	15.9
Ether extract	51.0	48.5
Crude protein	170.0	168.0
Crude fibre	52.0	47.0
aNDF	140.0	135.0
ADF	57.0	51.5
Starch	375.5	362.5
TMC, CFU/g^a^	< 100	< 100
Essential amino acids, g/kg		
Arginine	11.0	11.0
Histidine	4.1	4.1
Isoleucine	7.2	7.1
Leucine	12.6	12.8
Lysine	11.3	11.2
Methionine	2.5	2.6
Phenylalanine	8.0	8.0
Threonine	6.9	6.8
Tryptophan	2.2	2.3
Valine	7.9	7.7

*aNDF* Neutral detergent fibre (assayed with thermal-stable amylase and expressed inclusive of residual ash), *ADF* Acid detergent fibre, *TMC* Total mould count

All mycotoxins analysed were below the detectable levels: Aflatoxin B_1_, B_2_, G_1_ and G_2_ (< 1 μg/kg); Fumonisin B_1_ (< 125 μg/kg) and Fumonisin B_2_ (< 50 μg/kg) Deoxynivalenol (< 75.0 μg/kg), HT-2 Toxin (< 4.0 μg/kg), T-2 Toxin (< 4.0 μg/kg), Zearalenone (< 10.0 μg/kg) and Ochratoxin A (< 1.0 μg/kg)

^a^These values were log-transformed

### Statistical analysis

All data were tested for normality using the UNIVARIATE procedure of SAS software (package version 9.4) and residuals were inspected to confirm normality. The general linear model (PROC GLM) procedure of SAS was used to analyse sow body condition (BW and BF), the CATTD of nutrients, sow feed and energy intake and reproductive performance (gestation length, lactation length, piglets born (total, liveborn, stillborn), wean-to-oestrus interval and lactation efficiency). The model examined the effect of grain preservation method and used sow parity as a covariate. Piglet faecal scores during lactation were analysed using the linear mixed model procedure of SAS (PROC MIXED). The model included the maternal dietary effect and time and their associated interaction. Piglet growth parameters during lactation (litter weight, litter gain, piglet BW, piglet ADG and litter creep intake) and PW (pig ADFI, ADG and FCR) were analysed using PROC GLM. The data is presented as least-squares means with their standard errors of the mean (SEM). The faecal microbial populations were analysed using PROC GLIMMIX for nonparametric data. The model examined the maternal dietary effect and the faecal microbial results are presented using Benjamini–Hochberg adjusted *P*-values. The probability level that denoted significance was *P* < 0.05, with *P* values between 0.05 and 0.10 considered as tendencies.

## Results

### Grain and feed quality

Before grain preservation, the wheat samples had a moisture content 179.7 g/kg, a hectolitre weight of 71 kg/hL, and a thousand-grain weight of 47.4 g. For the barley samples, the moisture content was determined to be 182.1 g/kg, with a hectolitre weight of 62 kg/hL and a thousand-grain weight of 53.1 g.

Preserving grain with the OA mould inhibitor resulted in a lower DM content, grain pH and total mould count of preserved wheat and barley, while all other nutrients remained similar (Table 1). The levels of aflatoxin B_1_, B_2_, G_1_ and G_2_ (< 10 μg/kg), fumonisins B_1_ and B_2_ (< 1,000 μg/kg), DON (< 900 μg/kg), T-2 and HT-2 Toxins (< 50 μg/kg), ZEN (< 250 μg/kg) and OTA (< 50 μg/kg) were all below the detectable levels for both dried and preserved wheat and barley (not presented). Similarly, the preserved grain diet had lower DM, and mycotoxins in both diets remained undetected (Table 3).

### Sow body weight and back fat thickness

Sow body condition changes were not affected by diet during the experimental period. Sow BW changes and BF loss from d 100 of gestation to weaning were similar between dietary groups (Table S1).

### Coefficient of apparent total tract digestibility

The effect of grain preservation method on the CATTD of nutrients is presented in Table 4. Sows offered the preserved grain diet had increased CATTD of DM, OM, ash, N, aNDF and GE, as well as higher DE content compared to sows offered the dried grain diet (*P* < 0.05).

**Table 4:** The effect of maternal diet on the CATTD of nutrients, sow feed and energy intake, and lactation efficiency (least squares mean)

Maternal diet	Dried	Preserved	SEM	*P*-value
Digestibility coefficients				
Dry matter	0.842	0.856	0.003	**0.004**
Organic matter	0.869	0.882	0.003	**0.004**
Ash	0.424	0.459	0.016	**0.044**
Nitrogen	0.868	0.888	0.004	**0.002**
aNDF	0.510	0.562	0.014	**0.019**
Gross energy	0.847	0.864	0.003	**0.002**
DE content, MJ/kg^a^	13.62	13.82	0.055	**0.023**
Lactation feed intake				
ADFI, kg/d	7.01	7.02	0.023	0.764
DMI, kg/d	6.20	6.08	0.013	** < 0.001**
Average daily DE intake, MJ/d^b^	95.7	97.3	0.315	** < 0.001**
Average daily NE intake, MJ/d^c^	68.0	69.1	0.224	** < 0.001**
Lactation efficiency, g/MJ DE^d^	32.9	33.3	0.834	0.689

*aNDF* Neutral detergent fibre (assayed with thermal-stable amylase and expressed inclusive of residual ash), *ADFI* Average daily feed intake, *CATTD* Coefficient of apparent total tract digestibility, *DE* Digestible energy, *DMI* Dry matter intake, *NE* Net energy

^a^Calculated digestible energy, MJ/kg = (analysed dietary gross energy value, MJ/kg) × gross energy digestibility coefficient

^b^Calculated using analysed dietary DE values

^c^Calculated using calculated dietary NE values

^d^Calculated as: lactation efficiency = total litter weight gain from d 0 to 26 of lactation (kg) × 1000/sow lactation energy intake (MJ/DE)

### Lactation feed and energy intake and lactation efficiency

The lower DM of the preserved grain diet resulted in reduced dry matter intake (DMI) in sows offered the preserved grain diet compared to sows offered the dried grain diet (*P* < 0.05). However, sows offered the preserved grain diet had higher DE and NE intake compared to those offered the dried grain diet (*P* < 0.001). There was no effect of diet on lactation efficiency between treatments (Table 4).

### Sow reproductive performance

The reproductive parameters measured are presented in Table 5. The total number of piglets born (17.5 piglets) and number of piglets born alive (16.3 piglets) per litter were not influenced by maternal diet, nor was litter size at either d 10 (15.0 piglets) or d 26 postpartum (14.8 piglets). Consequentially, there was no maternal dietary influence observed on pre-weaning litter mortality. The length of gestation (116.0 d), lactation (25.9 d), and wean-to-oestrus interval (4.7 d) were similar between dietary groups (Table S1).

**Table 5:** The effects of maternal diet on litter size and pre-weaning piglet mortality (least squares mean)

Maternal diet	Dried	Preserved	SEM	*P*-value
No. of sows	20	20		
Parity	3.3	3.2		
Total born	17.7	17.3	0.571	0.569
Stillborn^a^	1.3	1.2	0.198	0.759
Litter size d 0	16.4	16.1	0.250	0.242
Litter size d 10^b^	15.0	15.0	0.280	0.956
Litter size d 26^c^	14.8	14.8	0.287	0.995
Pre-weaning mortality, %^d^	9.9	7.9	1.401	0.315

^a^Stillborn includes both stillborn and mummified piglets

^b^Creep introduction

^c^Weaning

^d^Pre-weaning mortality includes piglets that died from birth to weaning

### Progeny performance

The effect of maternal diet on progeny faecal scores and performance parameters is presented in Table 6. Piglets suckling sows offered the preserved grain diet had reduced faecal scores compared to those suckling sows fed the dried grain diet (*P* = 0.05). Litter weight and piglet BW during lactation were similar between dietary groups, and therefore, litter gain and piglet ADG throughout lactation were not affected by maternal treatment (*P* > 0.05). Pigs weaned from sows offered the preserved grain had improved ADG and FCR from weaning until slaughter compared to those weaned from sows offered the dried grain (*P* < 0.05). There was no maternal effect on progeny feed intake during lactation or PW (*P* > 0.05).

**Table 6:** The effect of maternal diet on offspring growth performance (least squares mean)

Maternal diet	Dried	Preserved	SEM	*P*-value
Pre-weaning performance				
No. of replicates	20	20		
Litter weight, kg				
d 0^a^	22.6	21.4	0.600	0.158
d10^b^	49.7	47.8	1.517	0.399
d 26^c^	106.3	106.1	2.182	0.938
Litter gain, kg				
d 0–10	27.2	26.6	1.166	0.712
d 10–26	56.6	58.1	1.280	0.385
d 0–26	83.7	84.7	1.840	0.710
Piglet body weight, kg				
d 0^a^	1.37	1.34	0.034	0.500
d 10^b^	3.27	3.17	0.076	0.384
d 26^c^	7.12	7.18	0.122	0.752
Average daily gain, kg/d				
d 0–10	0.19	0.19	0.006	0.678
d 10–26	0.24	0.25	0.006	0.299
d 0–26	0.22	0.23	0.005	0.445
Total creep intake, kg/litter	4.0	3.8	0.155	0.173
Lactation FS, score/litter^d^	1.75	1.38	0.134	0.050
Post-weaning performance				
No. of replicates	10	10		
ADG d 26–168, kg/d	0.78	0.82	0.115	0.048
ADFI d 26–168, kg/d	1.97	1.95	0.265	0.605
FCR d 26–168, kg/kg	2.52	2.38	0.042	0.049
Final BW, kg	118.0	123.6	2.431	0.123

^a^After cross-fostering

^b^Creep introduction

^c^Weaning

^d^Faecal score (FS) range: 1 = hard, firm faeces; 2 = slightly soft faeces; 3 = soft partially formed faeces; 4 = loose, semi-liquid faeces; and 5 = watery, mucous like faeces [44]

### Microbiological analysis

#### Bacterial richness and diversity

**Fig. 1:**
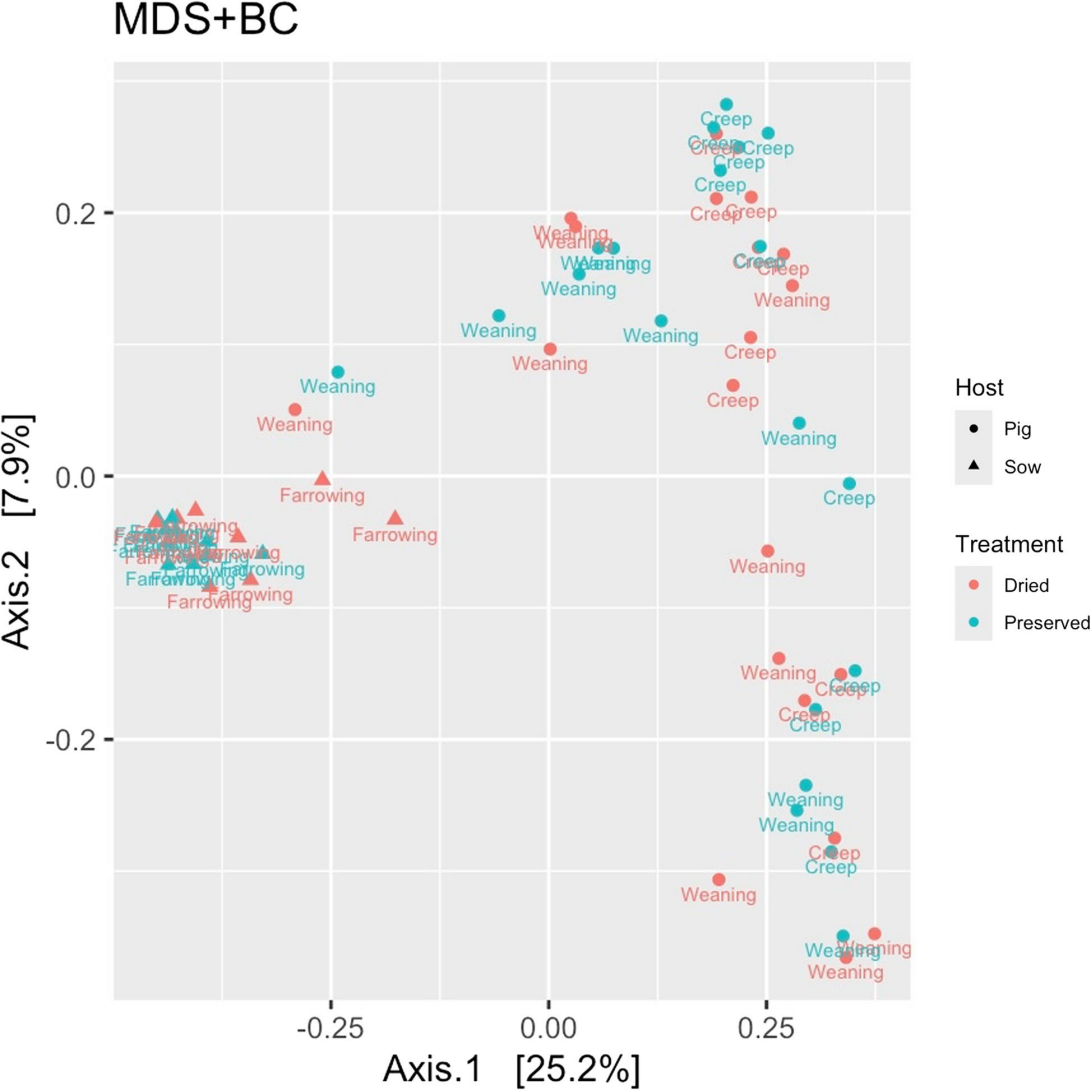
Bacterial beta diversity in sow faeces at farrowing and pig faeces on d 10 (creep) and d 26 (weaning), grouped by maternal diet, based on the Bray–Curtis distance matrix and visualised using multi-dimensional scaling

Beta diversity in sow faecal samples clustered separately from the beta diversity in piglet faecal samples (Fig. 1) however, there were no differences between groups based on PERMANOVA analysis through visualisation using the Bray–Curtis distance matrix and multi-dimensional scaling (*P* > 0.05). Similarly, there was no effect on the Observed, Shannon, Simpson, or Fisher indices of alpha diversity between sow dietary groups at farrowing or their offspring on d 10 and at weaning (Table S2).

#### Differential bacterial abundance

The effect of maternal diet on the bacterial abundance at phylum, family and genus level in sow faeces at farrowing, as well as their offspring on d 10 postpartum and at weaning, are presented in Tables 7, 8 and 9 and Tables S3–S5, respectively.

**Table 7:** The effect of maternal diet on the bacterial abundance at phylum level in sow and piglet faeces (%, least squares mean)

Maternal diet	Dried	Preserved	SEM	*P*-value
Sow at farrowing				
Firmicutes	70.42	68.12	2.632	0.545
Bacteroidetes	19.42	24.77	1.484	**0.021**
Proteobacteria	5.68	3.02	0.652	**0.012**
Actinobacteria	3.56	2.92	0.568	0.441
Piglet at d 10 postpartum				
Firmicutes	52.11	51.17	2.074	0.751
Bacteroidetes	41.01	43.11	1.873	0.436
Proteobacteria	3.76	2.15	0.488	**0.043**
Actinobacteria	2.56	2.00	0.435	0.380
Piglet at weaning (d 26)				
Firmicutes	53.42	56.87	2.489	0.341
Bacteroidetes	41.76	32.44	2.029	**0.006**
Actinobacteria	2.57	1.87	0.495	0.341
Proteobacteria	1.49	2.00	0.488	0.371

**Table 8:** The effect of maternal diet on the bacterial abundance at family level in sow and piglet faeces (%, least squares means)

Maternal diet		Dried	Preserved	SEM	*P*-value
**Phylum**	**Family**				
Sow at farrowing					
Firmicutes	Oscillospiraceae	4.21	8.15	0.776	**0.003**
	Lactobacillaceae	2.81	0.72	0.410	**0.031**
	Lachnospiraceae	2.93	0.41	0.371	**0.002**
	Christensenellaceae	2.44	4.41	0.579	**0.031**
Bacteroidetes	Rikenellaceae	8.74	13.15	1.041	**0.008**
Proteobacteria	Enterobacteriaceae	6.42	3.27	0.686	**0.006**
Piglet at d 10 postpartum					
Firmicutes	Oscillospiraceae	7.47	4.64	0.706	**0.008**
Bacteroidetes	Bacteroidaceae	6.00	8.88	0.784	**0.017**
	Muribaculaceae	10.48	3.39	0.733	**< 0.001**
	Tannerellaceae	1.18	2.69	0.473	**0.017**
Piglet at weaning (d 26)					
Firmicutes	Lactobacillaceae	7.38	10.98	1.032	**0.029**
Bacteroidetes	Prevotellaceae	8.65	4.87	0.855	**0.009**
	Muribaculaceae	5.43	8.80	0.904	**0.030**
	Rikenellaceae	23.53	15.74	1.468	**0.002**

**Table 9:** The effect of maternal diet on the bacterial abundance at genus level in sow and piglet faeces (%, least squares mean)

Maternal diet			Dried	Preserved	SEM	*P*-value
**Phylum**	**Family**	**Genus**				
Sow at farrowing						
Firmicutes	Oscillospiraceae	*Oscillibacter*	4.10	8.13	0.771	**0.002**
	Christensenellaceae	*Christensenella*	2.53	4.61	0.591	**0.026**
	Lactobacillaceae	*Lactobacillus*	2.93	0.74	0.451	**0.034**
Bacteroidetes	Rikenellaceae	*Anaerocella*	9.15	12.82	1.044	**0.024**
Piglet at d 10 postpartum						
Firmicutes	Oscillospiraceae	*Oscillibacter*	7.43	4.51	0.700	**0.008**
	Ruminococcaceae	*Sporobacter*	0.94	2.20	0.355	**0.026**
Bacteroidetes	Muribaculaceae	*Paramuribaculum*	7.22	2.75	0.627	**< 0.001**
	Bacteroidaceae	*Bacteroides*	6.01	9.12	0.790	**0.012**
	Muribaculaceae	*Muribaculum*	1.76	0.70	0.383	**0.033**
	Tannerellaceae	*Parabacteroides*	1.20	2.70	0.475	**0.003**
	Odoribacteraceae	*Butyricimonas*	0.70	1.80	0.315	**0.030**
	Bacteroidaceae	*Phocaeicola*	0.53	1.96	0.307	**0.012**
Piglet at weaning (d 26)						
Firmicutes	Lactobacillaceae	*Lactobacillus*	7.36	10.97	1.104	**0.029**
	Erysipelotrichaceae	*Holdemania*	0.80	3.10	0.442	**0.005**
	Lachnospiraceae	*Dorea*	1.70	0.37	0.319	**0.022**
Bacteroidetes	Rikenellaceae	*Alistipes*	16.05	11.66	1.237	**0.024**
	Prevotellaceae	*Prevotella*	8.77	4.81	0.859	**0.006**

#### Sow microbiota at farrowing

- Phylum: At farrowing, there were four bacterial phyla identified in sows, with Firmicutes being the most abundant (~ 69.27%), followed by Bacteroidetes (~ 22.10%), Proteobacteria (~ 4.35%) and Actinobacteria (~ 3.24%). Sows offered the preserved grain diet had an increased relative abundance of Bacteroidetes and a decreased abundance of Proteobacteria compared to sows offered the dried grain diet (*P* < 0.05).
- Family: Within the phylum Firmicutes, sows offered the preserved grain diet had an increased abundance of Oscillospiraceae and Christensenellaceae and a reduced abundance of Lactobacillaceae and Lachnospiraceae compared to sows offered the dried grain diet (*P* < 0.05). Within the phyla Bacteroidetes and Proteobacteria, the abundance Rikenellaceae and Enterobacteriaceae were both decreased in sows offered the preserved grain diet compared to those offered the dried grain diet (*P* < 0.01).
- Genus: Within the phylum Firmicutes, *Oscillibacter* and *Christensenella* were increased while *Lactobacillus* was decreased in sows offered the preserved grain diet compared to sows offered the dried grain diet (*P* < 0.05). *Anaerocella* (Bacteroidetes) was increased in sows offered the preserved grain diet compared to sows offered the dried grain diet (*P* < 0.05).

#### Piglet microbiota at d 10 postpartum

- Phylum: The predominant phyla in the piglet faecal samples at d 10 postpartum included Firmicutes (~ 51.64%), Bacteroidetes (~ 42.06%), Proteobacteria (~ 2.91%) and Actinobacteria (~ 2.28%). The relative abundance of Proteobacteria was decreased in piglets suckling sows offered the preserved grain diet compared to piglets suckling sows offered the dried grain diet (*P* < 0.05).
- Family: Within the phylum Firmicutes, the relative abundance of Oscillospiraceae was decreased in piglets suckling preserved grain-fed sows compared to those suckling dried grain-fed sows (*P* < 0.05). Within the phylum Bacteroidetes, Bacteroidaceae and Tannerellaceae were increased while Muribaculaceae was decreased in piglets suckling sows offered the preserved grain diet compared to piglets sucking sows offered the dried grain diet (*P* < 0.05).
- Genus: Within the phylum Firmicutes, the abundance of *Oscillibacter* was decreased while the abundance of *Sporobacter* was increased in piglets suckling preserved grain-fed sows compared to those suckling dried grain-fed sows (*P* < 0.05). Within the phylum Bacteroidetes, *Bacteroides*, *Parabacteroides*, *Butyricimonas* and *Phocaeicola* were increased while *Paramuribaculum* and *Muribaculum* were decreased in piglets suckling preserved grain-fed sows compared to the piglets suckling dried grain-fed sows (*P* < 0.05).

#### Piglet microbiota at weaning (d 26 postpartum)

- Phylum: At weaning, the predominant phyla in the piglet faeces were Firmicutes (~ 55.15%), Bacteroidetes (~ 37.10%), Actinobacteria (~ 2.22%) and Proteobacteria (~ 2.14%). Piglets from sows offered the preserved grain diet had reduced Bacteroidetes compared to piglets weaned from sows offered the dried grain diet (*P* < 0.01).
- Family: Within the phylum Firmicutes, the abundance of Lactobacillaceae was increased in piglets weaned from sows offered the preserved grain diet compared to piglets weaned from sows offered the dried grain diet (*P* < 0.02). Within the phylum Bacteroidetes, Muribaculaceae was increased while Rikenellaceae and Prevotellaceae were decreased in piglets weaned from preserved grain-fed sows compared to piglets weaned from dried grain-fed sows (*P* < 0.05).
- Genus: Within the phylum Firmicutes, *Lactobacillus* and *Holdemania* were increased while *Dorea* was decreased in pigs weaned from sows offered the preserved grain diet compared to pigs weaned from sows offered the dried grain diet (*P* < 0.05). Within the phylum Bacteroidetes, both *Alistipes* and *Prevotella* were decreased in piglets weaned from sows offered the preserved grain diet compared to piglets weaned from sows offered the dried grain diet (*P* < 0.05).

## Discussion

This study hypothesised that preserving cereal grains with an OA mould inhibitor could serve as an effective alternative to conventional grain drying. Additionally, it was hypothesised that incorporating OA-preserved grain into sow diets during late gestation and lactation would improve nutrient and energy digestibility, favourably alter the sow faecal microbiota, and ultimately benefit the microbial succession and growth performance of their offspring. The findings from this study revealed that sow and piglet performance during lactation were similar between dietary groups. However, pigs weaned from sows offered the preserved grain diet exhibited healthier faecal scores during lactation and enhanced growth and feed efficiency from weaning until slaughter. These improvements may be associated with the lower mould levels found in the preserved grain and alterations in microbial populations in both sows and piglets during lactation.

Maintaining high grain quality optimises animal health, welfare, and productivity [55]. Grains harvested with a higher moisture content are more susceptible to fungal growth and mycotoxin contamination [14, 56], which can negatively impact animal health and performance [57, 58]. In this study, the OA mould inhibitor effectively preserved the quality of wheat and barley at 180 g/kg moisture content, comparable to the quality achieved through drying to 140 g/kg. Although variations in DM were expected after storage, both preservation methods resulted in grains with similar chemical compositions and undetectable mycotoxin levels. The lower mould counts observed in the preserved grain highlight the efficacy of the mould inhibitor in mitigating the risks associated with fungal contamination, potentially preventing adverse effects from fungal secondary metabolites. Similar preservative properties of OA have been demonstrated in other studies [19, 28], with propionic-based products showing particular efficacy [27, 59]. Amid growing environmental and financial concerns regarding grain drying [16, 17, 60], this OA preservation technology offers a more energy-efficient alternative, while maintaining grain integrity and supporting herd productivity.

 Growing awareness of the environmental impact of feed production has intensified efforts to implement nutritional strategies that improve nutrient utilisation [61]. Dietary OA supplementation is widely recognised for enhancing nutrient digestibility across all stages of pig production [23, 62, 63]. In this study, significant improvements in the CATTD of DM, N, aNDF, and GE were observed in sows offered the preserved grain diet, highlighting this potential. Similar enhancements in nutrient digestibility have been reported in sows supplemented with OA additives during diet manufacture [34, 64, 65]. The acidifying effects of OA are often cited as a key mechanism behind improved nutrient digestibility, particularly in young pigs with lower acid secretion [25]. However, inconsistent pH modulation has been reported, especially with OA blends [66–68], and in older pigs with more mature digestive systems [69, 70]. Consistent with these observations, a recent study from our research group found that grower pigs (~ 22 kg) offered preserved grain exhibited improved ileal digestibility of nutrients without changes in gastric pH compared to those offered a dried grain diet [27]. While measuring ileal digestibility provides a more accurate determination of nutrient digestibility [71], collecting ileal digesta from sows was not feasible in this study.

Enhanced nutrient digestibility can play a crucial role in maintaining energy balance during lactation, by preventing excessive mobilisation of body tissue, and avoiding delays in the subsequent reproductive cycle [3, 72]. Thus, the lack of significant effects on BW change or BF loss in sows offered the preserved grain diet was unexpected, given the observed improvements in nutrient digestibility. Similar findings have been reported in sows of comparable parity and BW to those in this study when supplemented with OA additives [35, 64, 65]. In contrast, sows with lower parity and BW at farrowing that received OA blends have shown reduced BW and BF loss during lactation compared to non-supplemented sows [36]. This variability may be due to the variety of factors that can influence the benefits of OA, including the composition (acid type or salt form), concentration (individual acid, blend), supplementation duration, and the physiological state of the animal [22, 70]. These factors have also contributed to inconsistent responses in feed intake. While no obvious palatability issues were noted in this study, sows offered the preserved grain diet had lower DMI during lactation due to the lower DM content of the diet. This reduction was, however, compensated by improved GE digestibility, leading to increased DE content in the preserved grain diet and higher DE intake in sows throughout lactation.

Early acquisition of a stable and beneficial intestinal microbiota is essential for the health and development of suckling piglets [73, 74], as it supports growth, survival, and feed efficiency [75–77]. Dietary OA additives have been shown to enhance microbial diversity [78], and simultaneously inhibit pathogenic bacteria while promoting beneficial bacteria [79, 80]. In this study, no significant differences in microbial diversity were observed between dietary groups. However, the reduction in Proteobacteria, specifically *Enterobacteriaceae,* in sows offered the preserved grain diet is promising, given its association with gastrointestinal dysbiosis, inflammation, and opportunistic infections [81, 82]. The higher mould counts observed in the dried grains may have influenced microbial profiles, potentially facilitating the proliferation of taxa linked to dysbiosis, such as Proteobacteria. In contrast, the reduced levels of mould in the preserved grain , likely supported a healthier gut environment. While piglets initially share a microbial profile similar to their dam, this diverges within days to become individual-specific [83, 84]. Despite this divergence, piglets from sows offered the preserved grain diet also showed reduced Proteobacteria at d 10 postpartum, alongside lower faecal scores during lactation. These findings suggest that the maternal influence on early microbial establishment may have contributed to improved gut health [85].

At genus level, sows offered the preserved grain diet had increased abundances of *Anaerocella, Oscillibacter,* and *Christensenella* at farrowing. While swine-specific studies on *Anaerocella* are limited, *Oscillibacter* and *Christensenella* have both been reported to have strong anti-inflammatory properties, potentially offering additional gut health benefits [86, 87]. While these microbial changes were not directly reflected in their offspring, there were some beneficial shifts which may have promoted disease resistance and growth performance PW. At weaning, piglets from sows offered the preserved grain diet exhibited an increased abundance of *Lactobacillus* and a reduced abundance of *Alistipes* in their faeces. *Lactobacillus* can inhibit pathogenic bacteria and modulate the immune system [88], resulting in improved nutrient utilisation, growth, and feed efficiency [76]. In contrast, increased abundance of *Alistipes* has been linked to intestinal inflammation, gastrointestinal disorders and disrupted gut homeostasis [89–91]. The variations in microbial profiles observed in this study may have contributed to enhanced lifetime growth and feed efficiency in pigs weaned from sows offered the preserved grain diet, despite no differences in early-life performance during lactation. A similar ‘delayed’ response was observed in a study by Crespo-Piazuelo et al. [92], where piglets from sows supplemented with a probiotic showed no early-life performance improvements but later exhibited enhanced growth and feed efficiency from weaning to slaughter. The improved PW performance observed in the current study, coupled with the crucial role of the microbiota in physiological, nutritional, and immunological functions, emphasises the need for longitudinal studies to examine long-term changes in the intestinal microbiota and its implications on growth and feed efficiency. Exploring the inclusion of preserved grain in a combined maternal and offspring nutritional strategy could offer further benefits for piglet growth and health, but additional research is required to investigate its potential.

## Conclusion

In conclusion, this study highlights the potential for OA-preserved grains to effectively replace conventional dried grain as a fundamental feed ingredient in sow diets. The preserved grain not only exhibited lower levels of mould after storage but also enhanced the nutrient digestibility and energy intake in sows, compensating for the reduction in DMI. The favourable shifts in the gut microbiota observed in sows offered the preserved grain diet, and their piglets during lactation, may have contributed to enhanced gut health, improving piglet faecal scores during lactation, and increasing growth performance and feed efficiency from weaning to slaughter. As swine producers face increasing pressure to balance economic viability with environmental responsibility, the adoption of this preservation technology could play a role in shaping future sustainable pig production.

## Supplementary Information

Additional file 1: Table S1. The effects of maternal diet on gestation length, lactation length, wean to oestrus interval and sow body condition changes (least squares mean).

Additional file 2: Table S2. The effect of maternal diet on measures of alpha diversity (least squares mean).

Additional file 3: Table S3. The effect of maternal diet on the bacterial abundance (%) in sow faeces at farrowing (least squares mean).

Additional file 4: Table S4. The effect of maternal diet on the bacterial abundance (%) in piglet faeces on d 10 postpartum (least squares mean).

Additional file 5: Table S5. The effect of maternal diet on the bacterial abundance (%) in piglet faeces at weaning (d 26; least squares mean).

## Data Availability

All data generated and/or analysed during this study are available upon reasonable request from the corresponding author.

## Abbreviations

ADF: Acid detergent fibre
ADFI: Average daily feed intake
ADG: Average daily gain
AIA: Acid-insoluble ash
BF: Back fat
BW: Body weight
CATTD: Coefficient of apparent total tract digestibility
CFU: Colony-forming unit
CP: Crude protein
DE: Digestible energy
DM: Dry matter
DMI: Dry matter intake
DON: Deoxynivalenol
FCR: Feed conversion ratio
FS: Faecal scores
GE: Gross energy
N: Nitrogen
aNDF: Neutral detergent fibre assayed with a heat-stable amylase and expressed inclusive of residual ash
NE: Net energy
OA: Organic acid
OM: Organic matter
OTA: Ochratoxin A
OTU: Operational taxonomic unit
PW: Post-weaning
SEM: Standard error of the means
SID: Standard ileal digestible
TMC: Total mould count
ZEN: Zearalenone
