# Supplementary material for: Effects of organic acid-preserved cereal grains in sow diets during late gestation and lactation on the performance and faecal microbiota of sows and their offspring
Source: J Anim Sci Biotechnol. 2025 Mar 12;16:43. doi: 10.1186/s40104-025-01171-3 (PMC11899052; doi:10.1186/s40104-025-01171-3)
Supplement: Supplementary file 1 — Additional file 1: Table S1. The effects of maternal diet on gestation length, lactation length, wean to oestrus interval and sow body condition changes (least squares mean). [file 40104_2025_1171_MOESM1_ESM.docx]

**Table S1.** The effect of maternal diet on gestation length, lactation length, wean to oestrus interval and sow body condition changes (least square means ± SEM)

| Maternal diet^a^ | Dried | Preserved | SEM | P-value |
| --- | --- | --- | --- | --- |
| No of sows | 20 | 20 |  |  |
| Gestation length, days | 115.8 | 116.1 | 0.174 | 0.247 |
| Lactation length, days | 26.0 | 25.8 | 0.216 | 0.496 |
| Wean to oestrus interval, days | 4.6 | 4.7 | 0.204 | 0.848 |
| Sow BW at d 100 of gestation, kg | 271.2 | 273.6 | 4.468 | 0.699 |
| Sow BW at weaning, kg | 238.5 | 236.1 | 3.566 | 0.630 |
| BW change, kg | -32.7 | -37.5 | 2.596 | 0.187 |
| Sow BF at d 100 of gestation, kg | 16.0 | 16.1 | 0.308 | 0.875 |
| Sow BF at weaning, kg | 14.6 | 14.6 | 0.201 | 0.860 |
| BF change, mm | -1.4 | -1.5 | 0.145 | 0.565 |

BW, body weight; BF, backfat

^a^ Grain was either mechanically dried to a moisture content of 140 g/kg or preserved with an organic acid mould inhibitor at an inclusion rate of 4 g/kg and remained at 180 g/kg moisture content.
