# Supplementary material for: Effects of organic acid-preserved cereal grains in sow diets during late gestation and lactation on the performance and faecal microbiota of sows and their offspring
Source: J Anim Sci Biotechnol. 2025 Mar 12;16:43. doi: 10.1186/s40104-025-01171-3 (PMC11899052; doi:10.1186/s40104-025-01171-3)
Supplement: Supplementary file 2 — Additional file 2: Table S2. The effect of maternal diet on measures of alpha diversity (least squares mean). [file 40104_2025_1171_MOESM2_ESM.docx]

**Table S2.** The effect of maternal diet on indices of alpha diversity (least square means ± SEM)

| Maternal diet^a^ | Dried | Preserved | SEM | P-value |
| --- | --- | --- | --- | --- |
| Sow at farrowing |  |  |  |  |
| Observed | 50.70 | 51.70 | 2.92 | 0.774 |
| Shannon | 3.44 | 3.54 | 0.064 | 0.301 |
| Simpson | 0.95 | 0.97 | 0.006 | 0.214 |
| Fisher | 8.93 | 9.14 | 0.527 | 0.775 |
| Piglet at d 10 postpartum |  |  |  |  |
| Observed | 61.80 | 59.70 | 2.730 | 0.593 |
| Shannon | 3.51 | 3.58 | 0.069 | 0.483 |
| Simpson | 0.95 | 0.95 | 0.007 | 0.722 |
| Fisher | 11.35 | 10.89 | 0.617 | 0.615 |
| Piglet at weaning (d 26) |  |  |  |  |
| Observed | 54.20 | 54.60 | 2.302 | 0.904 |
| Shannon | 3.26 | 3.37 | 0.110 | 0.485 |
| Simpson | 0.92 | 0.93 | 0.016 | 0.511 |
| Fisher | 9.68 | 9.76 | 0.497 | 0.914 |

^a^ Grain was either mechanically dried to a moisture content of 140 g/kg or preserved with an organic acid mould inhibitor at an inclusion rate of 4 g/kg and remained at 180 g/kg moisture content.
