# Supplementary material for: Effects of organic acid-preserved cereal grains in sow diets during late gestation and lactation on the performance and faecal microbiota of sows and their offspring
Source: J Anim Sci Biotechnol. 2025 Mar 12;16:43. doi: 10.1186/s40104-025-01171-3 (PMC11899052; doi:10.1186/s40104-025-01171-3)
Supplement: Supplementary file 3 — Additional file 3: Table S3. The effect of maternal diet on the bacterial abundance (%) in sow faeces at farrowing (least squares mean). [file 40104_2025_1171_MOESM3_ESM.docx]

**Table S3.** The effect of maternal diet on the bacterial abundance (%) in sow faeces at farrowing (least square means ± SEM)

| Maternal diet^a^ | Dried^*^ | Preserved^*^ | SEM | P-value |
| --- | --- | --- | --- | --- |
| **Phylum** |  |  |  |  |
| Firmicutes | 70.42 | 68.12 | 2.632 | 0.545 |
| Bacteroidetes | 19.42 | 24.77 | 1.484 | **0.021** |
| Proteobacteria | 5.68 | 3.02 | 0.652 | **0.012** |
| Actinobacteria | 3.56 | 2.92 | 0.568 | 0.441 |
| **Family** |  |  |  |  |
| *Ruminococcaceae* | 29.07 | 24.65 | 1.638 | 0.07 |
| *Clostridiaceae* | 12.17 | 14.04 | 1.144 | 0.263 |
| *Prevotellaceae* | 10.71 | 9.35 | 1.001 | 0.350 |
| *Rikenellaceae* | 8.74 | 13.15 | 1.041 | **0.008** |
| *Enterobacteriaceae* | 6.42 | 3.27 | 0.686 | **0.006** |
| *Propionibacteriaceae* | 4.65 | 3.69 | 0.644 | 0.307 |
| *Lactobacillaceae* | 2.81 | 0.72 | 0.410 | **0.031** |
| *Oscillospiraceae* | 4.21 | 8.15 | 0.776 | **0.003** |
| *Erysipelotrichaceae* | 4.04 | 4.32 | 0.646 | 0.759 |
| *Muribaculaceae* | 3.48 | 4.59 | 0.634 | 0.232 |
| *Lachnospiraceae* | 2.93 | 0.41 | 0.371 | **0.002** |
| *Eubacteriaceae* | 2.65 | 1.82 | 0.471 | 0.236 |
| *Christensenellaceae* | 2.44 | 4.41 | 0.579 | **0.031** |
| *Acidaminococcaceae* | 1.28 | 1.40 | 0.366 | 0.827 |
| *Spiroplasmataceae* | 0.36 | 1.32 | 0.276 | **0.042** |
| *Hungateiclostridiaceae* | 0.21 | 0.42 | 0.176 | 0.431 |
| **Genus** |  |  |  |  |
| *Pseudoflavonifractor* | 19.77 | 16.61 | 1.347 | 0.115 |
| *Clostridium* | 12.91 | 14.58 | 1.172 | 0.328 |
| *Prevotella* | 11.25 | 8.76 | 0.998 | 0.097 |
| *Anaerocella* | 9.15 | 12.82 | 1.044 | **0.024** |
| *Propionibacterium* | 4.83 | 3.73 | 0.653 | 0.249 |
| *Sporobacter* | 4.83 | 3.50 | 0.643 | 0.163 |
| *Lactobacillus* | 2.93 | 0.74 | 0.451 | **0.034** |
| *Turicibacter* | 4.24 | 4.61 | 0.665 | 0.702 |
| *Oscillibacter* | 4.10 | 8.13 | 0.771 | **0.002** |
| *Ruminococcus* | 3.03 | 2.57 | 0.529 | 0.547 |
| *Eubacterium* | 3.00 | 1.96 | 0.495 | 0.159 |
| *Christensenella* | 2.53 | 4.61 | 0.591 | **0.026** |
| *Oscillospira* | 2.16 | 2.30 | 0.472 | 0.839 |
| *Phascolarctobacterium* | 1.40 | 1.44 | 0.377 | 0.942 |
| *Duncaniella* | 1.14 | 1.42 | 0.357 | 0.595 |
| *Muribaculum* | 1.14 | 1.63 | 0.370 | 0.362 |
| *Candidatus Armantifilum* | 0.61 | 1.11 | 0.290 | 0.250 |

^a^ Grain was either mechanically dried to a moisture content of 140 g/kg or preserved with an organic acid mould inhibitor at an inclusion rate of 4 g/kg and remained at 180 g/kg moisture content.
