# Supplementary material for: Effects of organic acid-preserved cereal grains in sow diets during late gestation and lactation on the performance and faecal microbiota of sows and their offspring
Source: J Anim Sci Biotechnol. 2025 Mar 12;16:43. doi: 10.1186/s40104-025-01171-3 (PMC11899052; doi:10.1186/s40104-025-01171-3)
Supplement: Supplementary file 4 — Additional file 4: Table S4. The effect of maternal diet on the bacterial abundance (%) in piglet faeces on d 10 postpartum (least squares mean). [file 40104_2025_1171_MOESM4_ESM.docx]

**Table S4.** The effect of maternal diet on the bacterial abundance (%) in offspring faeces on d 10 postpartum (least square means ± SEM)

| Maternal diet^a^ | Dried^*^ | Preserved^*^ | SEM | P-value |
| --- | --- | --- | --- | --- |
| **Phylum** |  |  |  |  |
| Firmicutes | 52.11 | 51.17 | 2.074 | 0.751 |
| Bacteroidetes | 41.01 | 43.11 | 1.873 | 0.436 |
| Proteobacteria | 3.76 | 2.15 | 0.488 | **0.043** |
| Actinobacteria | 2.56 | 2.00 | 0.435 | 0.380 |
| **Family** |  |  |  |  |
| *Lactobacillaceae* | 20.63 | 19.66 | 1.295 | 0.603 |
| *Rikenellaceae* | 14.33 | 15.41 | 1.113 | 0.503 |
| *Ruminococcaceae* | 11.90 | 12.30 | 1.004 | 0.782 |
| *Muribaculaceae* | 10.48 | 3.39 | 0.733 | **<0.001** |
| *Prevotellaceae* | 7.64 | 8.88 | 0.828 | 0.324 |
| *Oscillospiraceae* | 7.47 | 4.64 | 0.706 | **0.008** |
| *Bacteroidaceae* | 6.00 | 8.88 | 0.784 | **0.017** |
| *Lachnospiraceae* | 4.99 | 6.50 | 0.690 | 0.138 |
| *Eubacteriaceae* | 3.82 | 4.01 | 0.574 | 0.738 |
| *Propionibacteriaceae* | 2.59 | 2.00 | 0.437 | 0.352 |
| *Enterobacteriaceae* | 2.32 | 2.09 | 0.438 | 0.710 |
| *Clostridiaceae* | 1.80 | 1.85 | 0.393 | 0.927 |
| *Tannerellaceae* | 1.18 | 2.69 | 0.473 | **0.017** |
| *Odoribacteraceae* | 0.93 | 1.29 | 0.327 | 0.414 |
| *Christensenellaceae* | 0.79 | 0.38 | 0.258 | 0.210 |
| *Erysipelotrichaceae* | 0.27 | 0.14 | 0.130 | 0.493 |
| *Desulfovibrionaceae* | 0.25 | 0.33 | 0.155 | 0.712 |
| **Genus** |  |  |  |  |
| *Lactobacillus* | 20.78 | 19.46 | 1.295 | 0.477 |
| *Alistipes* | 13.64 | 14.67 | 1.086 | 0.509 |
| *Ruminococcus* | 6.63 | 6.31 | 0.743 | 0.762 |
| *Prevotella* | 7.77 | 8.53 | 0.843 | 0.314 |
| *Oscillibacter* | 7.43 | 4.51 | 0.700 | **0.008** |
| *Paramuribaculum* | 7.22 | 2.75 | 0.627 | **<0.001** |
| *Bacteroides* | 6.01 | 9.12 | 0.790 | **0.012** |
| *Eubacterium* | 3.82 | 4.04 | 0.572 | 0.787 |
| *Muribaculum* | 1.76 | 0.70 | 0.383 | **0.033** |
| *Propionibacterium* | 2.62 | 2.03 | 0.440 | 0.354 |
| *Lachnoclostridium* | 1.58 | 1.54 | 0.362 | 0.948 |
| *Clostridium* | 1.77 | 1.90 | 0.398 | 0.809 |
| *Blautia* | 1.56 | 2.65 | 0.415 | 0.084 |
| *Parabacteroides* | 1.20 | 2.70 | 0.475 | **0.003** |
| *Sporobacter* | 0.94 | 2.20 | 0.355 | **0.026** |
| *Anaerocella* | 0.76 | 0.69 | 0.251 | 0.849 |
| *Christensenella* | 0.70 | 0.31 | 0.241 | 0.206 |
| *Butyricimonas* | 0.70 | 1.80 | 0.315 | **0.030** |
| *Intestinimonas* | 0.62 | 0.70 | 0.241 | 0.818 |
| *Phocaeicola* | 0.53 | 1.96 | 0.307 | **0.012** |
| *Roseburia* | 0.21 | 0.26 | 0.146 | 0.801 |
| *Desulfovibrio* | 0.19 | 0.24 | 0.135 | 0.797 |
| *Pseudoflavonifractor* | 0.37 | 0.31 | 0.176 | 0.796 |
| *Holdemanella* | 0.16 | 0.14 | 0.110 | 0.905 |

^a^ Grain was either mechanically dried to a moisture content of 140 g/kg or preserved with an organic acid mould inhibitor at an inclusion rate of 4 g/kg and remained at 180 g/kg moisture content.
