# Supplementary material for: Effects of organic acid-preserved cereal grains in sow diets during late gestation and lactation on the performance and faecal microbiota of sows and their offspring
Source: J Anim Sci Biotechnol. 2025 Mar 12;16:43. doi: 10.1186/s40104-025-01171-3 (PMC11899052; doi:10.1186/s40104-025-01171-3)
Supplement: Supplementary file 5 — Additional file 5: Table S5. The effect of maternal diet on the bacterial abundance (%) in piglet faeces at weaning (d 26; least squares mean). [file 40104_2025_1171_MOESM5_ESM.docx]

**Table S5.** The effect of maternal diet on the bacterial abundance (%) in offspring faeces at weaning (d 26; least square means ± SEM)

| Maternal diet^a^ | Dried | Preserved | SEM | P-value |
| --- | --- | --- | --- | --- |
| **Phylum** |  |  |  |  |
| Firmicutes | 53.42 | 56.87 | 2.489 | 0.341 |
| Bacteroidetes | 41.76 | 32.44 | 2.029 | **0.006** |
| Actinobacteria | 2.57 | 1.87 | 0.495 | 0.341 |
| Proteobacteria | 1.49 | 2.00 | 0.488 | 0.371 |
| **Family** |  |  |  |  |
| *Rikenellaceae* | 23.53 | 15.74 | 1.468 | **0.002** |
| *Ruminococcaceae* | 15.29 | 15.26 | 1.309 | 0.986 |
| *Prevotellaceae* | 8.65 | 4.87 | 0.855 | **0.009** |
| *Lactobacillaceae* | 7.98 | 10.98 | 1.032 | **0.029** |
| *Lachnospiraceae* | 5.44 | 5.16 | 0.770 | 0.804 |
| *Muribaculaceae* | 5.43 | 8.80 | 0.904 | **0.030** |
| *Oscillospiraceae* | 5.73 | 7.16 | 0.851 | 0.250 |
| *Christensenellaceae* | 4.73 | 4.82 | 0.732 | 0.939 |
| *Eubacteriaceae* | 4.37 | 2.82 | 0.628 | 0.111 |
| *Propionibacteriaceae* | 2.85 | 1.73 | 0.499 | 0.147 |
| *Erysipelotrichaceae* | 1.81 | 2.44 | 0.489 | 0.368 |
| *Clostridiaceae* | 1.53 | 2.31 | 0.464 | 0.249 |
| *Enterobacteriaceae* | 1.42 | 1.97 | 0.475 | 0.361 |
| *Hungateiclostridiaceae* | 1.06 | 1.47 | 0.378 | 0.455 |
| *Bacteroidaceae* | 0.75 | 1.56 | 0.358 | 0.133 |
| *Acidaminococcaceae* | 0.62 | 0.55 | 0.254 | 0.853 |
| *Desulfovibrionaceae* | 0.23 | 0.49 | 0.200 | 0.372 |
| **Genus** |  |  |  |  |
| *Alistipes* | 16.05 | 11.66 | 1.237 | **0.024** |
| *Lactobacillus* | 7.36 | 10.97 | 1.104 | **0.029** |
| *Prevotella* | 8.77 | 4.81 | 0.859 | **0.006** |
| *Ruminococcus* | 6.49 | 7.74 | 0.888 | 0.334 |
| *Oscillibacter* | 5.30 | 7.05 | 0.826 | 0.156 |
| *Eubacterium* | 4.44 | 2.81 | 0.630 | 0.09 |
| *Paramuribaculum* | 3.92 | 4.88 | 0.698 | 0.346 |
| *Christensenella* | 3.91 | 3.62 | 0.647 | 0.754 |
| *Anaerocella* | 3.72 | 4.73 | 0.684 | 0.315 |
| *Phocaeicola* | 3.49 | 2.90 | 0.595 | 0.497 |
| *Propionibacterium* | 2.89 | 1.70 | 0.501 | 0.119 |
| *Gemmiger* | 1.92 | 1.64 | 0.445 | 0.664 |
| *Dorea* | 1.70 | 0.37 | 0.319 | **0.022** |
| *Blautia* | 1.69 | 1.17 | 0.397 | 0.375 |
| *Pseudoflavonifractor* | 1.49 | 0.51 | 0.322 | 0.063 |
| *Clostridium* | 1.37 | 2.27 | 0.446 | 0.178 |
| *Intestinimonas* | 1.02 | 1.53 | 0.374 | 0.348 |
| *Muribaculum* | 0.80 | 1.72 | 0.368 | 0.107 |
| *Holdemania* | 0.80 | 3.10 | 0.442 | **0.005** |
| *Roseburia* | 0.66 | 0.77 | 0.282 | 0.783 |
| *Bacteroides* | 0.66 | 1.60 | 0.346 | 0.091 |
| *Phascolarctobacterium* | 0.60 | 0.56 | 0.254 | 0.894 |
| *Duncaniella* | 0.44 | 0.22 | 0.189 | 0.437 |
| *Holdemanella* | 0.43 | 0.16 | 0.177 | 0.331 |
| *Sporobacter* | 0.35 | 1.12 | 0.275 | 0.092 |
| *Oscillospira* | 0.35 | 0.17 | 0.167 | 0.474 |
| *Lachnoclostridium* | 0.24 | 0.09 | 0.133 | 0.469 |
| *Desulfovibrio* | 0.20 | 0.50 | 0.192 | 0.305 |
| *Anaerobacterium* | 0.17 | 0.40 | 0.175 | 0.399 |
| *Peptococcus* | 0.15 | 0.07 | 0.106 | 0.614 |
| *Parabacteroides* | 0.09 | 0.37 | 0.151 | 0.272 |
| *Catenibacterium* | 0.02 | 0.02 | 0.049 | 0.993 |

^a^ Grain was either mechanically dried to a moisture content of 140 g/kg or preserved with an organic acid mould inhibitor at an inclusion rate of 4 g/kg and remained at 180 g/kg moisture content.
